# Convergence behaviours of energy series and GDP nexus hypothesis: A non-parametric Bayesian application

**DOI:** 10.1371/journal.pone.0271345

**Published:** 2022-08-04

**Authors:** Mihaela Simionescu, Wadim Strielkowski, Nicolas Schneider, Luboš Smutka

**Affiliations:** 1 Institute for Economic Forecasting, Bucharest, Romania; 2 Department of Trade and Finance, Faculty of Economics and Management, Czech University of Life Sciences Prague, Prague, Czechia; 3 Department of Agricultural and Resource Economics, University of California, Berkeley, Berkeley, CA, United States of America; 4 Department of Geography and Environment, The London School of Economics and Political Science (LSE), London, United Kingdom; J.C. Bose University of Science and Technology, YMCA, INDIA, INDIA

## Abstract

With the EU Green Deal initiatives, European members seek to launch the first climate neutral continent by 2050. This paper assesses the stochastic convergence of per capita energy consumption series for an illustrative sample of 15 EU countries with memberships prior to the 2004 enlargement, using data spanning the 1970–2018 period. Results from the confidence interval subsampling (asymmetric and equal-tailed) highlight that 11 out of the 15 EU series exhibit a long-run memory behaviour, while a diverging pattern was observed for the UK, Germany, Portugal, and Italy. Finally, per capita energy use series persist but fail to reveal one of the above dynamics for Ireland and Spain. Also, findings from the non-parametric Bayesian application (ANOVA/linear Dependent Dirichlet Process (DDP) mixture model) show how economic growth operates as a converging energy consumption-enabler over the long-run, from which the EU membership cannot be excluded. In particular, we highlight how the nature of energy-GDP hypotheses vary with the stochastic properties of energy use (converging behaviour with temporary shocks, converging pattern with permanent shocks, and diverging dynamic). Finally, our conclusions overcome the well-established development stage argument as we claim that countries with similar energy convergence patterns may need to adopt similar energy policies.

## 1. Introduction

While global warming is becoming a prominent issue of the modern world, energy reform can no longer disconnected from climate targets. Yet, it is clear that deploying secure supplies of electricity-based renewables may conflict with the needs from the booming energy demands recorded everywhere [1]. Long ago, seminal papers shed light on the dynamic linkages operating between energy consumption and economic growth [2–4]. Nonetheless, being endogenous by nature, those variables simultaneously respond to each other, thus constraining researchers to use error correction models accounting for the feedback structure of this relationship. Over time, a few consensus has been reached on how to reconcile power grids demand without jeopardizing long-run climate targets. Unless governments commit to drastic changes, global warming is indeed expected to induce massive land and biodiversity damages, threatening thus coastal populations and food security in the most vulnerable areas [5–7]. Nobuo Tanaka, Executive Director of the International Energy Agency (IEA, 2009) highlighted that “*Energy is at the heart of the problem–and so must form the core of the solution*”. But to implement changes in future energy paths, an in-depth understanding of the features of energy consumption series is required.

The development dilemma facing European Union (EU) countries remains how to reconcile the need for secure energy procurements for industrial and domestic purposes while avoiding unsustainable externalities [8, 9]. Following this view, both renewable and nuclear sources have emerged as a means of providing low-carbon alternatives to power-generation based-fossil fuels without boosting the energy dependence on foreign suppliers [10]. Naturally, enhancing the development of markets for low-carbon technology diffusion, and setting up better, more transparent, and more efficient market mechanisms are non-negligible steps [11]. But shifting to renewables and nuclear may induce a wide range of benefits including a massive supply to energy-intensive sectors, carbon savings, and limit the fossil price volatility traditionally facing domestic importers. So far, the structure of actual systems remains heavily fossil fuels-dependent, with conflicting economic, social, and industrial criteria. Thus, in a context of European Green Deal and a carbon-neutral target to be reached by 2050, the energy debate continues to sharply divide EU members.

The interest on assessing the stationary properties of energy consumption series lays in the far-reaching policy implications that unit root testing results confer. From a theoretical standpoint, a time-series is said stationary if its statistical properties (mean, variance, and autocorrelation) are constant over time. In a nutshell, if a series is stationary, one can predict that its properties will remain constant in the future, which turns highly useful when it comes to forecast and shocks and design policy responses. Said in other words, a process is considered to be stationary if its probability distribution is unchanged as the number of consecutive observations rises. This concept does apply to stochastic processes for which the data generation process does not vary over time. Naturally, one distinguishes between *strict* and *weak* stationarity (or stationarity), in the sense that the former refers to a stochastic equilibrium process *y*_*t*_ with similar realizations and distributions over different time intervals; whereas the latter designates processes for which the covariance between any two observations depends only on the length of time separating the observations [12]. While a weakly stationary process can also refer as a covariance stationary or wide-sense stationary time-series; one can measure how the concept of stationarity has played an important role in shaping the theory of stochastic processes and time-series analysis. From an empirical standpoint, if energy use is found to be stationary, then any shock affecting a country’s energy trends (i.e., emergence of a backstop technology reducing the marginal cost of a competitive resource; carbon tax set on producers; climate signal endorsed internationally with a few carbon leakages) will be temporary, and energy consumption is expected to shortly return to its normal and long-run equilibrium pace [13]. Conversely, in the presence of a unit root, a shock occurrence on energy use (i.e., electricity price shock) is more likely to have permanent effects, indicating that any shock (i.e., input price volatility) will be transmitted back to other macroeconomic variables [14].

Accordingly, assessing the patterns and properties of energy consumption series is of high interest. Despite the power mechanisms they drive, the stochastic convergence of energy use trends in "Old” EU Member States (OMS) known as EU-15 (i.e., Members of the EU area prior to the 2004 enacted “Eastern” Enlargement) remains pretty much overlooked. In this paper, we expect considerable structural differences between “Old” and “New” EU Member States (NMS) in terms of sustainable energy use and different policies should be considered in NMS to achieve within-EU convergence. However, one striking observation arising from a review of the literature is the lack of comparative analysis when dealing with integration properties of energy series, and OMS-related evidence. In general, previous assessments considered global homogenous EU samples and failed to provide country-specific insights. In doing so, previous papers generalized conclusions across panels exhibiting structural heterogeneities, with some risks of misleading policy implications that cannot be neglected. Also, the literature on stochastic convergence extensively employed univariate unit root testing procedures (Meng et al. (2013) for 25 OECD countries [15]; Fallahi et al. (2017) for 109 countries [16]; Liu and Lee (2020) for 107 countries) [17], but the use of stationary tests accounting for endogenously determined structural breaks remains not systematic. Here, we seek to fill those *two* above-mentioned gaps in a single manner.

Besides, in line with [1], we stress that the low power of conventional unit root tests in small samples can be overcome by using adequately fitted confidence intervals. This latter element represents the third competitive edge of this study. Analysing confidence intervals is thought to reorient the literature towards a better alternative than replicative “*black-box”* tests, which often avoid offering consistent and comparable results. Conversely, fitted confidence intervals can not only reveal the presence or absence of a unit root, but also capture the degree of persistence of this inference, bringing thus additional information for policy purpose. Finally, dynamic fluctuations in aggregate income are likely to influence the converging behaviours of energy series in a non-negligible manner. Therefore, we conduct the Panel Juodis, Karavias and Sarafidis [18] causality test for EU-15 countries (1970–2018), more adequate in relatively small samples; and attempt to capture the existence, nature, and direction of potential statistical causal linkages among economic and energy variables. Undoubtedly, there may exist a relation between the stochastic convergence of per capita energy series and the nature of the energy-GDP nexus hypothesis depicted by causality testing framework. While there is left for future research, we observe that no much less is known on this matter, which calls for a first inquiry.

Moreover, for data series suffering from large kurtosis, skewness and multimodality, standard parametric models are not recommended because of the potentially misleading results they can generate [19]. Therefore, without normal distribution in the data, a parametric approach may turn inconsistent because non-linearities may erode the relationship between variables, thus inducing misspecification issues. This bias, although pointed out in the early literature, remains overlooked by most of the recent Ordinary Least squares (OLS) regression studies on this topic, because basic preliminary statistics and hypotheses related to the distribution of residual error terms are often un-checked (independence, homoskedasticity, normality). For instance, if the errors do not follow a normal distribution, inaccurate p-values and confidence intervals for parameters are obtained. The errors autocorrelation and heteroscedasticity imply inefficient estimators for coefficients, biased estimators for parameters variance and incorrect values for t-statistic. Facing those challenges, this is where non-parametric Bayesian models find their contributions, and this represents the fourth novelty aspect of this paper. They are more operationally flexible than conventional econometric frameworks and allow for a wider range of data structures and distributions.

In sum, with the EU Green Deal initiatives, European members seek to launch the first climate neutral continent by 2050. In this paper, we assess the stochastic convergence of per capita energy consumption series for an illustrative sample of 15 EU countries, which encompasses economies with memberships prior to the 2004 Enlargement, and combined with data spanning the 1970–2018 period. Besides applying a stepwise integration property testing methodology, this research contrasts to previous ones as it offers a confidence interval subsampling (asymmetric and equal-tailed) to capture potential responses to shocks and converging dynamics among units. Results highlight that 11 countries out of the 15 EU series exhibit a long-run memory behaviour, while a diverging pattern was observed for the UK, Germany, Portugal, and Italy. Finally, per capita energy use series persist but fail to reveal one of the above dynamics in Ireland and Spain. Then, we elaborate ANOVA/linear Dependent Dirichlet Process (DDP) mixture model, which, to the best of our knowledge, is among the first applications of those models with real time series data on this precise topic. Results from which the non-parametric Bayesian model show how economic growth operates as a converging energy consumption-enabler over the long-run, from which the EU membership cannot be excluded. In particular, we highlight how the nature and directions of those causalities vary when groups of countries are stratified by stochastic convergence properties: converging pattern and temporary shocks, converging pattern and permanent shocks, and diverging pattern. Accordingly, adequate policy recommendations are proposed to orient per capita energy metrics towards a declining path.

Besides the Introduction, this paper is structured as follows: Section 2 presents the relevant literature. Section 3 displays the model set-up and econometric framework. In Section 4, one finds empirical results followed by a discussion and a comparison with past studies. Finally, Section 5 gives concluding remains, along with some policy implications, limitations of the study, and the pathways for further research.

## 2. Concise literature review

Over time, the literature has identified three convergence patterns: sigma-convergence, beta-convergence, and the stochastic convergence. Sub-cases of those classifications are: absolute convergence, real convergence, and conditional convergence. While the first one implies the achievement of the same equilibrium steady state for all countries in the sample; the second and third ones turn both necessary to achieve real convergence between per capita energy use and average value. However, the stochastic convergence may become conditional if each sample member converges to its own steady state as part of a model exhibiting a constant. On the other hand, long-run convergence is said to be achieved if per capita energy consumption series are stationary (they do not exhibit a unit root process) with a non-significant trend parameter. Conversely, lagged-type convergence or catching-up dynamic are achieved when per capita energy use series are integrated of order 0 or 1 (I(0) or I(1), respectively); and the trend is significant in the estimated equation [20]. In a nutshell, sigma-convergence indicates that disparities among various metrics are declining over time (GDP, unemployment, inflation, or energy use). In this context, one should observe that the standard deviation of each indicator records a significant decrease across periods. In neo-classical economic models and growth theories, two mechanisms lay under the phenomenon of beta-convergence: poor countries grow faster compared to rich ones (*i*) and the gap between rich and poor countries tends to decrease over time (*ii*). Thus, countries exhibiting a relatively low per capita energy use are expected to grow faster than those with larger energy metrics, because of a significant differential in energy intensities across economic structures (and sectors). As mentioned in [21], stochastic convergence is intrinsically linked to the temporal nature of a given shock on a certain country’s indicator, and its comparison with the average value of the same indicator for the entire sample. Accordingly, a shock to a certain indicator should return to its deterministic trend/mean.

In theory, stochastic convergence may imply that per capita energy use series are stationary, and reciprocally. According to [22], in the long-run, the prediction of the difference between per capita energy use in a country and the average value should converge towards a null value as the horizon forecast increases. From an empirical standpoint, most previous studies which combined convergence analysis with real energy consumption data relied on unit root testing frameworks. For instance, Meng et al. (2013) considered 25 OECD countries in the period 1970–2010 [15]; Fallahi et al. (2017) focused on 109 countries with data spanning 1971–2013 [16]; Liu and Lee (2020) explored the case of 107 countries with data from 1970 to 2010 [17]. If the null hypothesis of unit root is rejected, a converging pattern can be revealed for this indicator.

However, a well-known limit of unit root tests is the high sensitivity of outcomes to the time length of the sample period. For energy series covering a 30–50-year period, Smyth and Narayan (2015) showed that the empirical power of the time-series procedure may decline [23]. Thus, Fallahi and Voia (2015) proposed to rely on confidence intervals to alternatively [1]. While considering a sample of 25 OECD countries over the period 1960–2012, they showed how extracting information from confidence intervals can overcome conventional unit root challenges. In this paper, we follow those authors and combine both approaches together on an illustrative sample: the EU OMS group which are subject to common energy policy and to common energy targets in the European Green Deal. One guiding question is to determine whether a shock in energy consumption returns to its equilibrium path, or generates permanent effects.

Looking at empirical studies, while a range of relevant papers analyzed the property of integration specific to per capita energy use [24–31], only a few of them pushed the explicit aim of their study forward and investigated the consequential effect of stationary/non-stationary energy use series on the convergence pattern of countries.

One observes that most of previous studies are based on per capita energy consumption as indicator for which convergence is analysed [25, 32–37]. Often, previous empirical applications constrained the scope of their analyses to large sample of countries [16, 17, 35, 38, 39], OECD countries [1, 15, 40, 41] and U.S. states [36, 42]. Only a few of them paid an explicit attention to EU countries, but so far, we observe that energy indicators, sub-EU samples, and conclusions differ and often conflicts. Also, [43, 44] examined the convergence pattern of renewable energy consumption series in the EU-15 countries and 14 EU countries in the period 1990–2018 and 1990–2014, respectively. These last studies highlight the existence of convergence in renewable energy consumption, but overlooked the per capita energy use indicator, which highlights a critical gap in the literature. This is where this paper seeks to find its contribution. We notice that past papers extensively employed Lagrange Multiplier (LM) and Residual Augmented Least Squares-Lagrange Multiplier (RALS-LM) unit root tests with endogenously determined structural breaks. To the best of our knowledge, only [1] and [16] rooted their empirical assessment around the study of confidence intervals. Meanwhile, relevant triviale approaches combining energy-GDP-CO2 nexus assessments with the analysis of the integration properties in the series can be found in [45] for Israel; [46, 47], both for South Caucasus and Turkey; [48] for APEC countries; [49] for Italy. Also, additional panel and time-series evidence on the stationary properties of per capita energy consumption in EMU countries can be identified in [50, 51], respectively. Finally, an empirical investigation of the relationship between transportation infrastructure and economic development in China can be found in [52]. Also, an extensive review of the energy-GDP nexus literature can be found in [53], whereas [54] offers a state-of-the-art survey of the income-environment EKC domain. For more exhaustive information, we recommend these papers to the reader. Table 1 summarizes the main information on the empirical convergence literature.

**Table 1:** Summary of studies assessing the convergence properties of energy indicators.

Studies	Energy Indicators	Sample	Data Period	Econometric Method	Main Conclusion
[39]	PCRELC	96 countries	1980–2007	PA and NPA	Weak beta and sigma–convergence
[25]	PCEC	13 Pacific Island countries	1980–2005	PURT with SB	Strong Convergence
[35]	PCEC PCELC	108 countries	1971–2007	QR	Weak Convergence
[15]	PCEC	25 OECD countries	1960–2010	LM, RALS-LM	Strong Convergence
[33]	PCEC	ASEAN countries	1971–2011	LM unit root test	Strong Convergence
[1]	PCEC	25 OECD countries	1960–2012	ADF test; SCI	Convergence in 13 OECD states Persistent Convergence in per capita energy use in Spain, Greece, and Luxembourg
[16]	PCEC	107 countries	1971–2011	ADF test; SCI	Convergence in rich and well-developed countries
[34]	PCEC	seven sectors in Australia	1973–2014	Two-break and one-break LM, RALS-LM	Strong sectoral Convergence excepting transport sector
[38]	PCEC	109 countries and seven subsamples of countries	1971–2013	CIS for AR(q)	Convergence for all pairs of states
[42]	PCFEC	50 U.S. states	1960–2014	Two-break and one-break LM, RALS-LM	stochastic convergence in relative per capita fossil fuel use
[36]	PCEC	50 U.S. states	1970–2013	PA, NPA	No evidence of convergence (sigma, beta and stochastic convergence)
[41]	REC	27 OECD countries	1970–2011	PA, SPA	Convergence achieved by 14 countries
[37]	PCEC	OPEC countries	1970–2013	N-L test	Convergence in the case of all countries
[55]	PCCO2	44 developed and developing countries	1900–2015	RALS-LM unit root tests	Convergence
[33]	PCEC	Australian regions and 9 sectors	1990–2016	PSCCM	Multiple convergence clubs in 8 sectors; and similar energy consumption patterns in the case of states presenting similar characteristics
[56]	NGC	11 sectors in the U.S. states	January 1973—February 2017	UURT	convergence and persistence
[57]	ELC	Chinese regions	2000–2015	DDA	No evidence convergence
[58]	ELC	Indian states	1970–2015	PSCCM	Convergence observed for all Indian countries
[44]	REC	14 EU countries	1990–2014	System GMM method	Absolute and conditional convergence
[17]	EC	107 countries	1990–2015	SPSM	Convergence in 7 states out of 10 countries
[43]	REC	EU-15 countries	1990–2018	LM	Sigma, beta and stochastic convergence
[40]	REC	OECD countries	1965–2017	PF-TYA	Stochastic convergence in clean energy use for almost all states, with the exception of Canada, Denmark, Australia, Norway, Ireland, and Sweden
[59]	RELC	Brazil	1971–2014	N-L ARDL	The dynamic effects of growth, ecological footprint and globalization on electricity use are asymmetric

Source: our elaboration.

Notes: EC: total energy consumption; ELC: total electricity consumption; NGC: natural gas consumption; PCCO2: per capita carbon dioxide emissions; PCEE: per capita energy consumption; PCELC: per capita electricity consumption; PCFEE: per capita fossil energy consumption; PCRELC: per capita renewable electricity consumption; REC: renewable energy consumption; RELC: renewable electricity consumption. CIS for AR(q) confidence intervals for the sum of the AR(p) coefficients; DDA: Distribution dynamics analysis; RALS-LM: Lagrange Multiplier and Residual Augmented Least Squares-Lagrange Multiplier unit root test; N-L test: Non-Linearity test; NPA: Non-Parametric Approach; PA: Parametric Approach; PF-TYA: panel Fourier Toda-Yamamoto approach; PSCCM: Phillips-Sul club convergence method; PURT with SB: Panel Unit Root Test with Structural Break; QR: Quantile Regressions; SCI: Subsampling Confidence intervals; S-GMM: System-Generalized Method of Moments; SPA: Semi-parametric Approach; SPSM: Sequential Panel Selection Method; UURT: Univariate Unit Root Test.

## 3. Econometric framework

This study is rooted around two empirical hypotheses associated with specific methods:

**→H1**: *Persistent stochastic convergence in per capita energy use is present in some EU-15 and EU-13 countries*.
**→H2**: There is causality from *real economic growth to per capita energy use in the EU old member states*.

The converging dynamic is examined using a common unit root test such as Augmented Dickey-Fuller test (ADF) and specific confidence intervals, the latter being used as a better alternative for ADF test that has low power in the case of small samples. The nature and direction of causal relationships between economic growth and per capita energy use are investigated using ANOVA/linear Dependent Dirichlet Process (DDP) mixture model that is a Bayesian nonparametric (BNP) model with more levels. The advantages of BNP regression are related to flexibility, improvement in the precision of statistical inferences and credible data assumptions compared to parametric regression. Moreover, the BNP higher level model (known as BNP-HLM) considers random effect distributions and represents a flexible model-based cluster analysis. The construction of clusters is based on per capita energy use. In this study, three clusters of states are built: states having persistent shocks and converging pattern, countries without persistent shocks, but with converging behaviour, and states presenting diverging patterns. In this Section, a separate presentation of the methods is made. The first part of the section refers to confidence intervals, while the second one is dedicated to ANOVA/linear DDP mixture model.

A separate description of the methods is provided in this section: confidence intervals and ANOVA/linear Dependent Dirichlet Process mixture model.

### 3.1. The construction of confidence intervals (CIs)

The stationary character of a chronological series is confirmed if the upper limit of the confidence interval (CI) is less than 1, while unit root is detected in a series when the unit value is included. CIs construction starts from the highest coefficient of the autoregressive model of order p (AR(p)) denoted by *ρ*_*max*_ or from the sum of model’s parameters denoted by α. The latter approach makes the subject of the current study due to increase in the results’ accuracy [59]. The common CIs are the asymptotic ones. The lower and upper limits of the 90% CIs suppose the subtraction/adding the product between 1.645 and standard error of the root estimate. Discontinuity is encountered in the case of non-stationary series and roots close to 1 as [59] suggested. Given the limitations of this common approach, alternative methods are used. For example, ADF t-tests are inverted by Stock to construct the CI associated to the highest autoregressive root [60]. The maximum autoregressive root can be formulated as:

$$\rho_{max}=\frac{c}{T}+1$$ (1)

Where *T* refers to the sample size, c- deviation from unit root value. Hall shows that the percentile–t method considers the estimation of the autoregressive OLS parameter which represents the real value for evaluating sampling distribution of statistics and for the construction of CIs [61]. This method could not control for type I error. This limitation does not exist in the case of grid bootstrapping procedure with independent error as hypothesis [62]. Politis et al. proposed two approaches using subsampling method to withdraw the error independence [63]. The selection of the subsamples is made without replacement and the coefficients are estimated using OLS:

$$y_{t}=\mu +\beta t+\alpha y_{t-1}+\sum_{k=1}^{p-1}\gamma_{k}\Delta y_{t-k}+u_{t}$$ (2)

$$and\;\left\{ \begin{array}{c} t_{calculated}=b^{\frac{1}{2}}∙\frac{{\hat{\alpha }}_{b,m}-\hat{\alpha }}{{\hat{\sigma }}_{b,m}}\\ {\hat{\sigma }}_{b,m}=b^{\frac{1}{2}}∙se({\hat{\alpha }}_{b,m}) \end{array}\right.$$ (3)

where: ${\hat{\alpha }}_{b,m}$ is the OLS estimate associated to subsample *m;* b corresponds to the subsample size; m = 1,2,…,T-b+1; se refers to the standard error of the OLS estimate. The distribution associated to *t*_*calculated*_ is approximated by:

$$L_{b}(y)=\frac{1}{T-b+1}∙\sum_{m=1}^{T-b+1}1\;\{b^{\frac{1}{2}}({\hat{\alpha }}_{b,m}-\hat{\alpha })\leq y\}$$ (4)

Where $b^{\frac{1}{2}}$−normalizing constant; and the two-sided equal-tailed CIs for α are constructed as:

$$\left[\hat{\alpha }-\frac{1}{\sqrt{T}}∙c_{b,90\%},\hat{\alpha }+\frac{1}{\sqrt{T}}∙c_{b,10\%}\right]$$ (5)

Where *c*_*b*,90%_, *c*_*b*,10%_ are the 0.90 and 0.10 quantiles of the subsampling distributions. The results associated to two-sided equal-tailed CIs are pointwise consistent and two-sided symmetrical CIs are better because provide uniformly asymptotically valid results. The approximation of the empirical distribution is based on:

$$L_{b,|.|}(y)=\frac{1}{T-b+1}∙\sum_{m=1}^{T-b+1}1\;\{b^{\frac{1}{2}}(\left|{\hat{\alpha }}_{b,m}-\hat{\alpha }\right|)\leq y\}$$ (6)

Then, the Romano and Wolf algorithm is employed to determine the subsample size in four stages [64]:

- Stage 1: construction of 90% CI for α when b is chosen from the interval [*b*_*small*_, *b*_*big*_]; where $b_{small}=c_{1}∙\sqrt{T};\;b_{big}=c_{2}∙\sqrt{T};\;\frac{1}{2}\leq c_{1}\leq 1;\;{2\leq c}_{2}\leq 3$.
- Stage 2: calculation of the standard deviation associated to interval endpoints *I*_*b*,*lower*_ and *I*_*b*,*upper*_ for each b;
- Stage 3: selection of b value associated to the lowest standard deviation (b*);
- Stage 4: computation of the CI for the optimal value of b: [*I*_b*,*lower*_, *I*_b*,*upper*_].

Said in other words, the confidence intervals are built for the sum of coefficients in the autoregressive model of order p (AR(p)) known as α. The cumulative impulse response is computed for all horizons as $\frac{1}{1-\alpha }$. According to [65], there is a monotonic connection between α and cumulative impulse response. The use of α has few advantages: capacity to measure the shock using a single measure and quantification of spectrum at the null frequency. The traditional asymptotic confidence intervals are computed using the standard error: ($\hat{\alpha }-t∙\sigma$; $\hat{\alpha }+t∙\sigma$). The main limitation of this approach is given by the dependence of the distribution of OLS estimator of the unit value for α or not. This limitation is eliminated by considering in this study the subsampling procedure of Romano and Wolf [64] that solves the discontinuity of the distribution. From this point of view, valuable information on stochastic convergence is brought besides the unit root tests in terms of shocks persistence. These confidence intervals show the rejection or not of unit root, assess sampling uncertainty and indicate the consistent models with data [1]. An advantageous feature of this variant using subsampling method is results robustness to break points, because these points are dropped when CIs are constructed.

### 3.2. ANOVA/linear DDP mixture model

Another aim of this paper is to explain how the variable Y (per capita energy use) is related to x = (x_1_,x_2_,…,x_p_) (economic growth), and whether the nature of this relationship is influenced by the stochastic properties of energy consumption series. Here, we start from the data for $X={\left((1,x_{i}^{T})\right)}_{nx(p+1)}$ and *y* = (*y*_1_,…,*y*_*n*_)^*T*^. *i* = 1,…,*n* is indexes the individual data observations. Under the hypothesis of a constant (1) and p covariates, *x* = (1, *x*_1_,…,*x*_*p*_)^*T*^. The regression coefficients are *β* = (*β*_0_, *β*_1_,…,*β*_*p*_)^*T*^, *β*_0_ is the constant and *β*_1_,…,*β*_*p*_ represent the slopes that are seen as effects associated to those p covariates. *σ*^2^ is the dispersion associated to errors *ε*_*i*_. The normal distribution of parameters mean *μ* and dispersion *σ*^2^ is written as *N*(*μ*, *σ*^2^). Normal p.d.f. (bell-shaped) is written as $n(y|\mu ,\sigma^{2})=\frac{1}{\sigma \sqrt{2\pi }}exp(-\frac{{\left(y-\mu \right)}^{2}}{2\sigma^{2}})$. The likelihood function of y given x with coefficients *ϑ* = (*β*, *σ*^2^) is represented by *f*(*y*_*i*_|*x*; *ϑ*). The approach starts from a linear model:

$$y_{i}=x_{i}^{T}\beta +\varepsilon_{i},\;\varepsilon_{i}\to N(0,\sigma^{2}),i=1,2,\ldots ,n$$ (7)

This model could be alternatively expressed as:

$$f(y_{i}|x_{i};\vartheta )=n(y_{i}|x_{i}^{T}\beta ,\sigma^{2}),i=1,2,\ldots ,n$$ (8)

OLS estimates are computed as: $\hat{\beta }={\left(X^{T}X\right)}^{-1}X^{T}y,{\hat{\sigma }}^{2}=\frac{1}{n-p-1}\sum_{i=1}^{n}{\left(y_{i}-x_{i}^{T}\;\hat{\beta }\right)}^{2},y={\left(y_{1},\ldots ,y_{n}\right)}^{T}$ and $X={\left((1,x_{i}^{T})\right)}_{n*(p+1)}$. Looking at the general functional form of the BNP model:

$$f(y|x;\;\vartheta )=\int f(y|x,\tau ,\theta )dG_{x}(\theta )=\sum_{j=1}^{\infty }f(y|x,\tau ,\theta_{j}(x)\omega_{j}(x)$$ (9)

Where {*f*(.|*x*, *τ*, *θ*)}: (*θ*, *τ*)}∈Θ corresponds to the kernel densities (selected famility of parametric densities); *ω*_*j*_(*x*) are mixing weights whose sum is 1 for each *x*∈ϰ; *δ*_*θ*(*x*)_(.) probability measure having the property to degenerate at *θ*(*x*); *τ*- additional coefficients that are not included in the mixture; {*ω*_*j*_(*x*)}_*j*_, {*θ*_*j*_(*x*)}_*j*_ infinite collections of processes with indexation after ϰ. The prior distribution for coefficients should be specified in the Bayesian density regression, *x*∈ϰ:

$$\vartheta =\left(\tau ,{\left(\omega_{j}(x),\theta_{j}(x)\right)}_{j}\right)$$ (10)

Dependent Dirichlet Process (DDP) is employed in most of the Bayesian density regressions [73]. DDP prior is *G*_*x*_~*DDP*(*α*, *G*_0*x*_). The random distribution is represented as: $G_{x}=\sum_{j=1}^{\infty }\omega_{j}(x)\delta_{\theta_{j}(x)}(.)$. The stick-breaking weights are calculated by [75] such as:

$$\left\{ \begin{array}{c} \omega_{j}(x)=v_{j}(x)\prod_{l=1}^{j-1}(1-v_{j}(x)),j=1,2,\ldots \\ v_{j}∼Q_{j},v_{j}:\varkappa \to [01,]\\ \theta_{j}(x)∼{ind\;G}_{0x}(the\;atoms) \end{array}\right.$$ (11)

ANOVA/linear DDP model is constructed as a mixture model with mixing distribution by Iorio et al. [66]:

$$\left\{ \begin{array}{c} G∼Stick-Breaking\;({\left(a_{j},b_{j}\right)}_{j},G_{0})\leq >G_{X}(\theta )∼ANOVA-DDP\;({\left(a_{j},b_{j}\right)}_{j},G_{0})\\ G_{x}(\theta )=\sum_{j=1}^{\infty }\omega_{j}(x)\delta_{\theta_{j}(x)}(\theta )\\ \theta_{j}(x)=x^{T}\beta_{j}\\ \beta_{j}|\mu \end{array}\right.$$ (12)

Where *T*~*iid G*_0_ = *N*(*μ*, *T*); Normal kernel *n*(*y*|*θ*, *σ*^2^). In this study, grouping variable (multilevel) presents three variants: 1—for states having persistent shocks and converging pattern, 2 –for countries without persistent shocks, but with converging behaviour, and 3—for states presenting diverging patterns. The countries allocated to each cluster are determined after the studying stochastic convergence based on ADF unit test and asymmetric CIs.


$${{(y}_{i(h)})}_{i(h)}^{n_{h}}|X_{h}∼f(y_{h}|X_{h}),h=1,\ldots ,N_{h}$$

$$f(y_{h}|X_{h})=\sum_{j=1}^{\infty }\{\prod_{i(h)=1}^{n_{h}}n(y_{i(h)}|x_{i(h)}^{T}\beta_{j},\sigma^{2})\}\omega_{j}$$

$$\omega_{j}=v_{j}\prod_{l=1}^{j-1}\left(1-v_{l}\right)$$

$$v_{j}|\alpha ∼Be(1-a,b+aj)$$

$$\sigma^{2}∼IG(\frac{a_{o}}{2},\frac{a_{o}}{2})$$

$$\beta_{j}|\mu ,T∼N(\mu ,T)$$

$$\mu ,T∼N(\mu |0,r_{0}I_{p+1})IW(T|p+3,s_{0}I_{p+1})$$

The weight of 1 is assigned to any observation. A number of 4000 Monte Carlo samples are drawn from 20000 samples (we except the burn-in of 2000 samples and thinning from Monte Carlo samples). The optimal MC mixing of parameter is CUSUM = 0.5, where 0 < CUSUM < 1 (CUSUM median-centred) [67]. The dependent variable is the relative per capita energy use (PCEU) for any country *i*. This variable is calculated as natural logarithm of the ratio (per capita energy use in a state *i*/ the average per capita energy use for overall group:

$$y_{it}=ln\;(\frac{{PCEU}_{it}}{\bar{{PCEU}_{t}}}).$$

The source of data for per capita energy use in the period 1970–2018 (unit of measurement: kg of oil equivalent per capita) is the World Bank database. Energy use refers to use of primary energy before transformation to other end-use fuels, which is equal to indigenous production plus imports and stock changes, minus exports and fuels supplied to ships and aircraft engaged in international transport. The explanatory variables are the logarithm of GDP per capita (constant 2010 US$) from the World Bank and the EU membership (values: 0 if a country was non-EU member at moment *t* and 1 if the country is EU member at time *t*). GDP per capita is computed by dividing the gross domestic product to mid-year population. GDP in constant 2010 U.S. dollars is computed by the summing up the gross value added of resident producers at national level with products’ taxes and subtracting subsidies that are not in the products’ value. The descriptive statistics are displayed in Table 2 for few years in the analyzed period. The lowest value for the period 1970–2018 in the case of PECU was observed in Portugal in 1970, while the highest level of PCEU was reached by Luxembourg in the same year. In terms of economic growth, the highest increase was observed Luxembourg in the last year of the period, while Portugal registered the minimum value in 1970. A clear increasing trend in the economic growth was observed for all the countries in the period 1970–2018 which supports the economic progress.

**Table 2:** Descriptive statistics of PCEU series with and logarithmic-transformed GDP per capita.

Year	PCEU series				logarithm transformed GDP per capita series			
	Mean	Standard deviation	Minimum value	Maximum value	Mean	Standard deviation	Minimum value	Maximum value
1970	3477.132	2618.793	663.8615	12106.15	9.8393	0.359	9.077	10.476
1980	3770.874	2037.253	1022.464	9774.651	10.112	0.313	9.423	10.660
1990	3951.466	1755.432	1680.962	8874.105	10.342	0.334	9.721	11.096
2000	4221.496	1426.68	2390.098	7676.837	10.582	0.349	9.975	11.445
2010	4232.515	1656.019	2222.63	8329.478	10.673	0.358	10.021	11.561
2018	3704.963	1323.273	2131.682	6548.406	10.699	0.405	9.999	11.586

Source: our elaboration.

In the period 1970–2010, the per capita energy use registered an ascending tendency, but with a decreasing rate for most of the states. PCEU increased by 8.45% in 1980 compared to 1970. A lower increase was observed in 1990 compared to 1980 (4.79%). A higher rate of increase (6.83%) was reached in 2010 with respect to 2000, while a very low growth was observed in 2010 compared to 2000 (0.26%). A sudden decrease appeared in 2018 corresponding to a lower PCEU in 2018 compared to 2010 by 12.43%.

Besides, skewness, kurtosis, Shapiro-Wilk test and IQR statistics are provided in Table 3. As stated earlier, when data series suffer from relatively large kurtosis, skewness and multimodality, standard parametric models are not recommended because of the potentially inconsistent estimates they can generate [19]. Therefore, without a normal distribution in the data, a parametric approach may turn inconsistent because non-linearities may erode the relationship between variables, thus inducing potential misspecification issues. This bias, although pointed out early in the literature, remains overlooked by a wide range of parametric OLS regression studies on this topic, because basic preliminary statistics and hypotheses related to the distribution of residual error terms are often un-checked (independence, homoskedasticity, normality). Thus, parametric methods may be unfitted for the current data structure, which then justifies the use of an alternative approach. Accordingly, in this paper, we follow Mohammadi and Ram [36] and rely on a non-parametric approach, thought to present more suitable features to those statistical conditions. In general, we justify the adoption of a non-parametric approach by the fact that it represents a relevant alternative when dealing with relatively small sample sizes (*i*); it relies on less constraining statistical assumptions regarding the properties and the shapes of the data (in particular, when dealing when non-normally distributed data) (*ii*); it is particularly relevant when the data series to be assessed are inherently in ranks or categories (nominal, ordinal, interval or the data which has outliers) (*iii*); whereas interpretations are more straightforward and thus less likely to be misleading (*iv*) [68]. Hence, since they do not assume that the structure of the model is fixed, non-parametric tests present the advantage of reducing the risk of misleading results if the model specification does not capture the characteristic of the dynamic of interest (here energy consumption) [69]. In a pioneer contribution, Breitung stressed that using test statistics that do not require the specification of the short-run dynamics or the estimation of nuisance parameters present a high empirical potential, also because the variance ratio statistic for a nonparametric unit root test can be generalized to test hypotheses on the cointegration [70]. Besides, the non-parametric approach turns attractive when samples are large, in which small deviations from the underlying (parametric) assumptions may have a substantial effect on the behavior of the parametric test statistic. The reason is that the short-run component does not affect the asymptotic null distribution of the test statistic. Thus, the test is robust against deviations from the usual assumption of linear short-run dynamics. when the sample size is large, there is reason to expect that the random walk component dominate the sampling behavior of the nonparametric test statistic and the asymptotic theory provides a reliable approximation to the actual null distribution. Nonetheless, non-parametric tools may suffer from size problems that can be observed in the standard fractional seasonal variance ratio tests, especially under extreme patterns of heteroskedasticity [71]. Thus, Gög̃ebakan and Eroglu very recently proposed a new non-parametric seasonal unit root testing framework that is robust to periodic non-stationary volatility in innovation variance [72].

**Table 3:** Jarque-Bera test, Shapiro-Wilk test and IQR statistics.

Country	Jarque-Bera test				Shapiro-Wilk W test				IQR	
	PCEU		GDP		PCEU		GDP		PCEU	GDP
	Adjusted chi-square statistics	p-value	Adjusted chi-square statistics	p-value	z stat.	p-value	z stat.	p-value		
Austria	14.19	0.002	13.47	0.0012	4.787	0.0000	2.567	0.00512	0.05475791	0.58357239
Denmark	23.15	0.0000	12.79	0.0017	0.757	0.22461	2.567	0.00513	0.24542317	0.4641552
Belgium	19.34	0.0000	13.70	0.0011	4.655	0.0000	2.305	0.01059	0.1754565	0.54605293
Finland	20.03	0.0000	12.47	0.0020	5.589	0.0000	3.002	0.00134	0.1227460	0.60681915
Germany	26.45	0.0000	9.32	0.0095	3.542	0.0002	2.473	0.00670	0.29313955	0.4618454
Greece	10.26	0.0000	2.38	0.3045	3.406	0.00033	0.612	0.27035	0.28654146	0.3161068
Ireland	17.25	0.0000	14.84	0.0006	1.892	0.02925	2.408	0.00803	0.13367125	1.2259045
Luxembourg	4.45	0.1079	26.82	0.0000	2.656	0.00396	3.268	0.00054	0.27900875	1.1458893
Netherlands	15.84	0.0000	24.51	0.0000	0.640	0.26102	3.071	0.00107	0.08763433	0.65129662
Portugal	11.26	0.0036	9.66	0.0080	3.539	0.00020	3.782	0.00008	0.57906622	0.60283089
Spain	18.67	0.0000	22.20	0.0000	1.998	0.02286	3.132	0.00087	0.24708903	0.68936348
Sweden	15.65	0.0000	14.28	0.0008	1.666	0.04790	2.291	0.01099	0.12857389	0.55375671
France	3.28	0.1936	9.31	0.0095	2.017	0.02186	2.572	0.00505	0.03435816	0.51522255
Italy	17.45	0.0000	6.03	0.0489	0.532	0.29747	3.851	0.00006	0.08658901	0.36733246
UK	4.61	0.0999	18.36	0.0001	2.945	0.00162	2.773	0.00278	0.18978087	0.6071949

Source: own calculations

Jarque-Bera test, Shapiro-Wilk W test and IQR are computed for PCEU and logarithm of GDP per capita in Table 3. Excepting Luxembourg and France, the data series for PCEU do not follow a normal distribution as Jarque-Bera test indicates at 10% significance level. All the countries excepting Greece present non-normal distribution for logarithm of GDP per capita at 10% significance level. According to Shapiro-Wilk test, excepting Denmark, Italy and Netherlands, data series for PCEU present non-normal distribution at 5% significance level. The data series for logarithm of GDP per capita do not follow normal distribution for all countries excepting Greece at 5% significance level.

According to these results, non-parametric approach is a better alternative to parametric methods and a Dirichlet Process mixture model is constructed in the next section.

## 4. Empirical results and discussion

The analysis of stochastic convergence for the relative PECU is based on unit root tests (Augmented Dickey-Fuller (ADF) test and Zivot-Andrews test that considers structural break. The Table 4 presents the results based on these tests. The unit root assumption is rejected at 10% significance level only for Netherlands, Ireland, and Austria. Therefore, convergent pattern in PECU can be observed only in these countries.

**Table 4:** Results of univariate ADF and structural break-accounting Zivot-Andrews tests and subsampling confidence intervals for EU-15 countries (1970–2018).

Country	Computed ADF statistics	t-Statistic (intercept and trend)	Breaking point (year)	Lag length based on Akaike information criterion	90% two-sided equal-tailed Cis	90% two-sided symmetrical Cis
**Austria**	-4.188244*	-4.8923***	2000	1	(0.924,1.005)	(0.893,1.001)
**Denmark**	-3.045078	-4.2503	1990	1	(0.559,0.804)	(0.459,0.667)
**Belgium**	-1.632411	-3.3177	1990	2	(0.892,1.055)	(0.792,0.999)
**Finland**	-2.082472	-3.7138	2007	1	(0.992,1.005)	(0.847,0.987)
**Germany**	-1.857837	-3.0554	1988	1	(0.765,1.101)	(0.722,1.044)
**Greece**	-2.610545	-4.5722	2009	1	(0.880,0.980)	(0.884,0.976)
**Ireland**	-3.246908*	-4.8389***	2007	9	(0.934,0.979)	(0.922,0.976)
**Luxembourg**	-2.678845	-3.071404	2002	3	(0.822,1.006)	(0.805,0.956)
**Netherlands**	-3.451407*	-4.6954***	2002	1	(0.630,0.792)	(0.622,0.788)
**Portugal**	-0.828548	-2.361861	2005	1	(0.957,1.022)	(0.933,1.007)
**Spain**	-1.813588	-3.946280	2007	1	(0.945,1.056)	(0.922,0.988)
**Sweden**	-2.073744	-3.658247	1982	1	(0.763,1.033)	(0.676,0.934)
**France**	-1.497246	-3.235966	2005	1	(0.882,1.002)	(0.872,0.982)
**Italy**	-0.629846	-3.854280	2007	1	(0.901,1.067)	(0.892,1.043)
**UK**	-1.207710	-3.460227	2006	1	(0.892,1.238)	(0.873,1.198)

Source: our elaboration.

Notes

***p<0.01

**p<0.05

*p<0.10.

For these states with convergence in PECU, we should check if the trend significant. In the ADF equation, we check the significance of the trend for Netherlands, Ireland, and Austria and time trend proves to be significant for all states at 0.1 significance level. In conclusion, Austria, Ireland, Netherlands present catching-up type or lagging-behind convergence in per capita energy use. This result is confirmed for Austria by [1], when the authors analyzed the convergence in per capita energy use in 25 OECD countries during 1960–2012.

The Akaike information criterion (AIC) is employed to select the lag length that corresponds to the lowest value for AIC. Table 4 also describes the 90% confidence intervals for the sum of autoregressive coefficients corresponding to each level of relative PECU: symmetrical two-sided and equal-tailed CIs. These CIs were computed for each value. Thus, all the roots of the models are represented by real numbers. Although the ADF test indicates a converging pattern for Austria, confidence intervals suggest a diverging trend. The capacity to manage the low misspecifications in the errors’ auto-correlation and better subsampling performance make the symmetrical CIs more accurate and tighter compared to equal-tailed CIs. The converging pattern was not revealed in the case of Belgium, Luxembourg, Finland, Sweden, Spain, and France according to equal-tailed CIs. The results are not affected by any break points in the data series. In the case of symmetrical CIs, subsampling method was used, and a confidence interval is constructed for each subsample using a similar manner. This sampling method is repeated until the end of units. If subsamples exhibit break points, only inferior and superior limits of the approximated distribution are affected, but these are excluded when the 90% CIs are elaborated. However, the results of Zivot-Andrews test are also included. Results based on symmetrical CIs are exhaustively explained:

- *First*, the upper bounds of the intervals are compared to 1. The superior limits of the CIs are lower than 1 for 10 states: Belgium, Greece, Denmark, Netherlands, Finland, Ireland, Spain, France, Luxembourg, and Sweden. The PECU is stationary in this case and the conclusion is that a converging pattern is observed for these countries at 10% significance level.
- *Second*, the lower bounds are compared to the value 0.9. For an inferior limit greater than 0.9, the data series is very close to non-stationary property which I equivalent with a very persistent effect of one shock. In this case, the lower bounds are greater than 0.9 for Spain and Ireland which implies a persistent shock.
- *Third*, the intervals length is observed. Good accuracy is obtained for narrow intervals indicate good precision of the results. The quite narrow intervals obtained in this case confirm the results precision.

Results might be sensitive to the information criterion used to select the optimal lag. Therefore, two more criteria are used for selection: Schwarz information criterion (SIC) and Hannan-Quinn information criterion (HQ). A single difference was observed between the section based on AIC and those based on HQ and SIC. This exception is represented by Belgium for which lag length is 1 when HQ and SIC are considered, while AIC suggested the value 2. The 90% symmetrical and equal-tailed CI for this country in the case of lag length equalling 1 are (0.898,1.034), respectively (0.903,0.992). According to these results, Belgium presents a converging behaviour per capita energy use with a persistent shock.

To conclude, results based on confidence intervals, ADF and Zivot-Andrews tests indicate stationarity in relative PCEU in almost all the EU-15 countries, the exception being given by Portugal, Germany, Italy and UK that present a diverging behaviour. The PCEU in the UK reduces, while the indicator increases faster than average growth rate in Italy, Portugal, and Germany. The stationarity in relative PCEU does not suppose compulsory stationarity in the PCEU of a state if the average PCEU in the old member states is not stationary. The ADF unit root test is applied again on mean of PECU (the calculated statistic is -1.223) which supposes non-stationary data series. According to HQ and SIC, the optimal lag is 1, while AIC suggests the value 9. The 90% symmetrical confidence intervals corresponding to lag 9 [(0.829, 0.945)] and to lag 1 [(0.841, 0.979)] suggest stationarity in national PECU. These results could be compared to other studies to point out differences and similarities, although it is worth noticing that sample size and data periods differ in previous papers. In other words, a comparative analysis with the relevant literature should be rooted around the emergence of converging and diverging behaviours and the potential presence of persistent shocks. Table 5 puts into perspective the present findings and those of previous studies; stratified by stochastic behaviour of energy series.

**Table 5:** Classification of previous studies’ results and comparison our stochastic convergence outcomes for PECU.

Evidence of diverging PECU series (in line with the present study)	Evidence of converging PECU series (in line with the present study)	Evidence of persistent shocks in PECU series (in line with the present study)
UK ([1, 16]); Portugal ([16]); Germany ([1, 16]); Italy ([15])	Denmark, Belgium, Finland, Ireland, Luxembourg, Netherlands ([1, 15], [16])	Spain ([1, 16])

Source: our elaboration.

Table 5 suggests that diverging behaviours were observed in Italy by Meng et al. [15] (as part of a OECD-based analysis over the period 1960–2010) and for Germany, UK and Portugal by Fallahi et al. [16] (same sample structure, but over a shorter period (1971–2011)). Unlike other studies ([1, 16]), states like Netherlands, Ireland, France and Denmark presented a persistent shock. These empirical results present far-reaching information for policy purpose. For countries with converging behaviour, energy conservation and energy demand policies used to control climate changes will have short term impact. Government interventions are not necessary to design economic policies controlling for energy consumption. For countries displaying persistent shocks, environmental public regulation may not represent the most adequate solution. However, for countries with diverging pattern, government initiatives may need to identify, target, and regulate any unwanted deviations. The comparisons between EU-15 countries (Old Member States) and EU-13 countries (New Member States) in terms of stochastic convergence in PECU are insightful. However, because of data availability constraint, most of the data related EU-13 countries are hardly complete and balanced before 1990. Therefore, we conduct a distinctive and separate analysis over the period 1990–2018 to offer the tools for a reliable comparison between Old and New Member States and give a robustness check of the findings. Results displayed in Table 6 suggest that there persists a converging pattern for Baltic States and Malta at 5% significance level for both confidence intervals; whereas a converging pattern for Cyprus is depicted only when 90% two-sided equal-tailed Cis are considered. In general, such shock remains persistent for Estonia and Cyprus only, while remaining countries present diverging patterns.

**Table 6:** Results of univariate ADF and structural break-accounting Zivot-Andrews tests and subsampling confidence intervals for EU-13 countries (1990–2018).

Country	Computed ADF statistics	t-Statistic (intercept and trend)	Breaking point (year)	Lag length selection (k)	90% two-sided equal-tailed CIs	90% two-sided symmetrical CIs
**Croatia**	-1.802321	-1.423651	2010	1	(0.923,1.121)	(0.902,1.099)
**Cyprus**	-3.137885**	-3.409342	2012	1	(0.916,0.999)	(0.909,1.001)
**Czechia**	-0.957947	-3.167290	2014	2	(0.923,1.002)	(0.916,1.001)
**Estonia**	-2.974214***	-4.903646**	2009	1	(0.947,0.976)	(0.945,0.998)
**Hungary**	0.473974	-1.948608	2014	2	(0.958,1.04)	(0.943,1.03)
**Latvia**	-3.336821**	-4.973583**	2002	2	(0.877,0.923)	(0.866,0.917)
**Lithuania**	-2.970534***	-6.321057*	2009	1	(0.678,0.834)	(0.643,0.818)
**Malta**	-4.167361*	-10.28280*	2003	1	(0.703,0.902)	(0.699,0.899)
**Poland**	-1.870831	-2.923777	2007	2	(0.935,1.22)	(0.993,1.18)
**Romania**	-1.976372	-4.877105**	1998	2	(0.955,1.06)	(0.938,1.05)
**Slovakia**	0.571046	-2.361698	1999	1	(0.894,1.08)	(0.922,1.1)
**Slovenia**	-2.353605	-2.949797	2014	1	(0.992,1.033)	(0.99,1.028)

Source: our elaboration.

Notes

***p<0.01

**p<0.05

*p<0.10.

According to Table 7, similar results were obtained for EU-15 countries in the period from 1990 to2018 compared to 1970–2018, except for Austria for which stationary is checked and the shocks are persistent. Moreover, the shock becomes persistent in Finland and Greece.

**Table 7:** Results of ADF and Zivot-Andrews tests and subsampling confidence intervals for EU-15 countries (1990–2018).

Country	Computed ADF statistics	t-Statistic (intercept and trend)	Breaking point (year)	Lag length selection (k)	90% two-sided equal-tailed CIs	90% two-sided symmetrical CIs
**Austria**	-3.320147**	-5.105607**	2000	4	(0.955,0.993)	(0.909,0.992)
**Denmark**	0.013002	-7.373111*	2005	1	(0.665,0.922)	(0.569,0.872)
**Belgium**	-0.556629	-3.226712	2005	2	(0.902,1.01)	(0.852,1.02)
**Finland**	-2.370012	-4.080323	2007	1	(0.958,1.012)	(0.903,0.992)
**Germany**	-2.106576	-3.647368	2012	1	(0.805,1.11)	(0.831,1.05)
**Greece**	-2.908650	-3.177635	2009	1	(0.903,0.988)	(0.892,0.989)
**Ireland**	-2.677303*	-5.179670***	2004	5	(0.955,0.998)	(0.926,0.987)
**Luxembourg**	-2.005903	-5.391137*	2003	3	(0.933,1.01)	(0.876,0.976)
**Netherlands**	-0.395103	-7.016475*	2005	1	(0.706,0.842)	(0.678,0.722)
**Portugal**	-2.104754	-3.103090	2005	1	(0.988,1.025)	(0.954,1.017)
**Spain**	-2.709218***	-3.590342	2007	1	(0.976,1.073)	(0.929,0.995)
**Sweden**	-1.156402	-3.691065	2006	1	(0.789,1.038)	(0.702,0.998)
**France**	-0.502364	-5.195218**	2005	1	(0.882,1.002)	(0.898,0.991)
**Italy**	0.449558	-1.845391	2008	1	(0.952,1.023)	(0.891,1.015)
**UK**	0.682012	-5.467818	2007	1	(0.853,1.118)	(0.923,1.239)

Source: our elaboration.

Notes

***p<0.01

**p<0.05

*p<0.10.

The optimal lag length selection is based on the information provided by the Akaike Information Criterion (AIC).

The optimal lag length selection is based on the information provided by the Akaike Information Criterion (AIC).

For the two samples (New Member States and Old Member States), four states with converging pattern and persistent shocks emerge (Estonia, Cyprus vs. Ireland, Spain, Finland, and Greece). Overall, 10 out of 15 countries in the EU-15 present convergence in per capita energy use, while only 4 out of 13 countries in the new member states fulfil convergence requirements. The covariance matrix of OLS estimator for the regression coefficients appears to be inconsistent in the case of mis specified model mainly due to heteroscedasticity bias. One way to deal with this issue is to use White’s sandwich covariance matrix estimator [73]. Also, it is worth notifying that recent studies employed an alternative Bayesian approach that could be extended to include informative prior distributions. In general, a high volume of data and non-trivial prior specifications are required to build Dependent Dirichlet process (DDP) infinite-mixture regression models.

The Dirichlet process is known as the most important process prior that is used in the Bayesian non-parametric statistics, because of its flexibility in approximation under any type of probability law and exchangeability in data [74]. Before the estimation of Dirichlet Process mixture model, the value of subjective priors should be mentioned taken the values proposed by Karabastos [75]. Subjective priors might be constructed using specific methods like scoring and elicitation. Moreover, Swartz showed that the ignorance prior converging to zero is the common non-informative prior used in many studies from literature [76]. When the ignorance prior converges to zero, then estimated value of the mean in the population equals the sample mean. We considered the fact that expected prior probability for any interval should be proportional to the length of the interval. The procedure follows five general stages: specification of priors, consideration of 50,000 MC samples, generation of MCMC samples from the model’s posterior distribution.

- The first coefficient of Stick Breaking (SB) prior (0≤*a*<1): Dirichlet Process selected by a = 0;
- The second coefficient of SB prior (b). If a = 0, then b = α is the precision parameter associated to Dirichlet Process. In this particular situation, b = 1;
- The prior SB baseline dispersion of model parameters (*r*_0_ = 10);
- The prior SB baseline scale of model parameters (*s*_0_ = 10);
- The prior SB baseline shape and rate ($\frac{a_{0}}{2},\frac{a_{0}}{2}$) of the error variance *σ*^2^. *a*_0_ = 5 is considered.

In this case, the grouping variable refers to type of stochastic convergence and takes three values corresponding to the 3 groups of countries that were previously identify based on unit root tests and confidence intervals:

1. Countries with converging behaviour and no persistent shocks (Belgium, Austria, Greece, Luxembourg, Denmark, Finland, Sweden, Netherlands, and France);
2. Countries with converging trend and persistent shocks (Spain and Ireland);
3. Countries with diverging patterns (Portugal, UK, Germany, and Italy).

First, the variables scaling is made before estimation to have null mean and unit variance. The Intra Class Correlation coefficient (ICC) in Table 8 suggests the proportion of the variation in the per capita energy use determined by between-groups heterogeneity. In this case, in average, 60.3% of the variation in PCEU is explained by heterogeneity between the groups of countries.

**Table 8:** Posterior summary statistics of estimates.

Parameter	Mean	Standard deviation	25%	75%
*β*_0_ (sample)	0.612	0.314	0.061	0.830
*β*_*economic*_*growth*_(sample)	0.080	0.995	0.077	0.082
*β*_*EU_membership*_(sample)	0.414	0.878	0.279	0.637
*σ* ^2^	0.822	0.125	0.753	0.902
$\beta_{economic_{growth}}(group=1)$	0.066	0.223	0.054	0.102
$\beta_{economic_{growth}}(group=2)$	0.075	0.237	0.058	0.094
$\beta_{economic_{growth}}(group=3)$	0.003	0.776	-0.002	0.007
*β*_*EU_membership*_(group = 1)	0.242	0.554	0.207	0.335
*β*_*EU_membership*_(group = 2)	0.302	0.655	0.289	0.422
*β*_*EU_membership*_(group = 3)	0.117	0.335	0.112	0.129
ICC	0.603	0.137	0.505	0.723
Reliability *β*_0_*R*	0.789	0.033	0.756	0.826
Reliability *β*_*economic_growth*_*R*	0.825	0.045	0.799	0.834
Reliability *β*_*EU*_*membership*_R	0.827	0.045	0.785	0.842

Source: our elaboration.

If all countries are computed and analysed all together, the 75% posterior intervals do not include the value 0 which suggest that EU membership and growth are significant predictors and causes for PCEU. Conversely, group level-analyses indicate that economic growth causes PCEU only for countries with converging pattern, regardless the shock persistence. Interestingly, the nature and direction of this causality is not significant for countries presenting diverging PCEU patterns. The EU membership had a significant and positive impact on PCEU in all groups of countries due to growth spillover and convergence dynamics. In the period 2010–2018, the energy consumption reduced, but further comprehensive measures are necessary. In particular, economic policies are especially needed in locations where PCEU presents long-term effect due to shocks in GDP per capita. Linking with the literature, a causal relationship running from growth to energy consumption was confirmed by Zachariadis for a few G7 countries [77], but this excludes the development stage argument which stresses that the nature of this relationship may be determined by the per capita income and industrial levels [78].

To offer a robustness check, the Juodis, Karavias and Sarafidis test is applied to assess causal relationships for each panels [18]. This method presents the advantage of allowing for heterogenous slope coefficients and cross-sectional dependence among the series, which is likely to be the case here. Results are shown in Table 7. This procedure is conducted on stationary data. According to Breitung test, the data for economic growth and PCEU are stationary only in the first difference in all groups. For data in level (non-transformed) of GDP, we have the following statistics for which p-value is higher than 0.1: group 1(6.6579), group 2 (5.9878), group 3(7.7723). For the data in the first level in the case of growth, the statistics are the following: group 1 (-6.7401), group 2 (-6.0234), group 3 (-5.9984). The data for PCEU are stationary in first-difference levels. The statistics for data in level and first differences are provided in brackets for each group: group 1(2.4145/-8.846), group 2 (2.0234/-8.9934), group 3 (1.9745/-9.0374). Therefore, this panel causality testing method is performed on first-difference series, as shown in Table 9.

**Table 9:** Results of the panel Juodis, Karavias and Sarafidis (JKS, 2021) causality test for EU-15 countries (1970–2018).

Group of countries	Null hypothesis (H_0_)	Half-Panel Jackknife Wald test statistic	p-value	Results for the Half-Panel Jackknife estimator	
				Coefficient	p-value
**Countries with converging behaviour, without persistence of shocks (Austria, Denmark, Belgium, Finland, Greece, Luxembourg, Netherlands, Sweden and France)**	ΔEconomic growth does not Granger-cause ΔPCEU	4.3099877	0.0379	0.0056	0.038
	EU membership does not Granger-cause ΔPCEU	4.0334544	0.0404	0.0034	0.04
	ΔPCEU does not Granger-cause Δeconomic growth	1.2589589	0.2618	9.504721	0.262
	ΔPCEU does not Granger-cause EU membership	0.01021015	0.9195	-0.0005512	0.920
**Countries with converging pattern and persistent shocks (Ireland and Spain)**	ΔEconomic growth does not Granger-cause ΔPCEU	20.405322	0.0000	3.550631	0.0000
	EU membership does not Granger-cause ΔPCEU	5.7685096	0.0163	0.0180185	0.016
	ΔPCEU does not Granger-cause Δeconomic growth	2.2220403	0.1361	-0.0418445	0.136
	ΔPCEU does not Granger-cause EU membership	1.993455	0.2023	0.0034758	0.202
**Countries with diverging patterns (Germany, Portugal, UK and Italy)**	ΔEconomic growth does not Granger-cause ΔPCEU	0.15505544	0.6938	0.0050906	0.694
	EU membership does not Granger-cause ΔPCEU	20.037867	0.0000	5.97654	0.0000
	ΔPCEU does not Granger-cause Δeconomic growth	5.4456311	0.0196	-0.09712	0.02
	ΔPCEU does not Granger-cause EU membership	15.234565	0.0000	0.0444655	0.0000

Source: our elaboration.

Notes

***p<0.01

**p<0.05

*p<0.10.

The optimal lag length selection is based on the information provided by the Akaike Information Criterion (AIC).

According to Juodis, Karavias and Sarafidis causality test, changes in economic growth and EU membership drive variations in PCEU in countries with converging behaviour. On the other hand, only EU membership seem triggering PCEU in states displaying a divergence in per capita use. However, PCEU variations cause growth and EU membership in the EU-15 countries with divergence. On the one hand, JKS results only partially corroborate those of Simionescu and Schneider [79] as they failed to reject the null hypothesis of no causality for a share of top nuclear energy consumption countries. On the other hand, because series are pooled all together before estimation, those panel evidence are generalized at the sample level, which cannot go without some sources of bias. Indeed, our findings may suggest that EU countries with comparable stages of development may adopt similar energy policies [80]. Naturally, some researchers have gone one step further by conducting a large panel analysis on various income groups. However, these investigations only considered electricity series. For instance, Apergis and Payne categorized 88 countries into four distinct panels following the World Bank income classification [81]. Findings suggested that the nature of the electricity consumption-economic growth nexus is indeed determined by the country’s stage of development, but only partially. While low-income country evidence supported the “growth hypothesis” (emphasizing that electricity plays a crucial role in the development process), as income grows, the interdependence between electricity consumption and economic growth may shift towards a feedback structure, so that any fluctuation in GDP may directly transmit back to changes in power use series. Thus, once aggregate incomes reach high levels, the growth of the economy may be rather driven by the domestic consumption of energy. This could relevantly explain the validation of the “conservation hypothesis” for countries with converging pattern and persistent shocks (Ireland and Spain) and countries with converging behaviour, without persistence of shocks (Austria, Denmark, Belgium, Finland, Greece, Luxembourg, Netherlands, Sweden, and France). According to this view, our results may be generalizable to some OECD and EU-27 countries with important policy insights.

Nonetheless, other relevant studies underlined that energy-GDP nexus cannot be generalized to countries sharing a comparable stage of development. Akinlo argued that African countries should not adopt the same energy conservation measures, since time-series causality analysis for 11 sub-Sahara African economies gave conflicting conclusions [82]. In the same way, Acaravci and Ozturk supported different energy-growth relationships while considering 19 developed European countries, which is in line with our sub-group-level insights [83]. By implication, this literature predicates that each country may develop its own energy strategy given its peculiar economic characteristics. More recently, Aydin and Esen shed light on another potential determinant of the energy-growth link: the level of energy intensity [84]. They estimated a threshold level of energy intensity, above which energy consumption significantly hinders growth. Knowing that the energy-GDP linkage can be asymmetric, researchers should pay more attention to efficiency gains while assessing the relationship among energy and growth. Going beyond the standard development stage argument supported in Magazzino and Schneider [53], this paper claims that countries with similar convergence patterns in energy consumption series may adopt similar energy policies. Those two latter determinants being potentially interconnected. There is left for future research.

## 5. Conclusions and policy implications

Extracting value from the stochastic properties of per capita energy use series may offer non-negligible information for decarbonization and climate policies purposes. This study investigated the stochastic convergence of per capita energy consumption series in 15 EU countries, with yearly information covering the 1970–2018 period. We employ a stepwise conventional integration property testing method and propose a confidence interval subsampling (asymmetric and equal-tailed, following [1]) to capture potential responses to shocks and converging dynamics among units. One advantage of this latter approach compared to standard unit roots tests is the ability to identify the persistence of a shock and measuring sampling uncertainty, thus overcoming the low power of ADF unit root test. Then, we combine ANOVA/linear Dependent Dirichlet Process (DDP) mixture model with real time series data. Among the explicit aims of the paper, one of them seeks to highlight how the nature and directions of energy-GDP linkages vary across stochastic convergence properties groups (converging pattern and temporary shocks, converging pattern and permanent shocks, and diverging pattern). Parametric ADF results and 90% CIs suggested that there exists a stochastic convergence in per capita energy consumption for 11 countries belonging to the EU-15 (Austria, Belgium, Denmark, Greece, Finland, Luxembourg, Netherlands, Spain, Ireland, Sweden, and France). However, the diverging pattern reported in Portugal, Italy, and Germany may find explanation within the faster PCEU growth they have recorded compared to its average trend, while the UK exhibits a declining trend, mainly due to energy efficiency gains.

Accordingly, adequate policy recommendations are proposed to orient per capita energy metrics towards a declining path. In general, one important novelty of this paper is that our results go beyond the standard development stage argument and claim that countries with similar convergence patterns in energy consumption series may adopt similar energy policies. For EU countries presenting converging pattern, shocks due to policies for demand management generate volatile responses and local interventions may, under some conditions, be inadequate. Therefore, common EU regulation (i.e., those imposed at the central EU level in a form of the “top-bottom” approach) may be relevant. However, in the case of dynamic shocks displaying long-run effects and inferences, more suitable energy policies incorporating country-specific features and constraints are needed. For economies with diverging patterns, local and national-oriented responses may generate larger substantial effects than broad, homogenous, and generalized EU level policies, with only a few framework’s margin for country-level adaptation and flexibility. On the path towards the European Green Deal, countries may have a better benefit in implementing policies based on their particular characteristics. All in all, internalizing energy poverty reforms within future energy planning may reconcile both economic and Sustainable Development Goals (SDGs) together.

Therefore, additional efforts are required and should be rooted around energy efficiency, low-carbon technology diffusion, and fossil fuels taxation, for which carbon tax should take a centre stage [85]. While the anticipation effects of fossil resource producers cannot be neglected by environmental planners, the effective impacts of “*Green Paradox*” mechanisms on climate targets tend to be over-estimated and might become smaller under reasonable assumptions, circumstances, and sectors, it does not mean that the effectiveness of climate policies can be disconnected from the dynamic supply response from resource owners, as shown in the pioneer paper of Hotelling [85]. Instead, upcoming environmental planning should internalize the anticipating nature of fossil resource owners through: strong and timely signals accompanied with a relatively short implementation lag (< ten years) (*i*); Credible policies able to avoid cumulatively higher emissions induced by the failing expectation of imminent regulations of producers (*ii*); the setting of an optimal carbon tax (starting high, rising fast) deemed appropriate to balance the marginal costs and benefits of fossil energy use. While it has been shown that the preannouncement of taxes that start low and rise slowly does not significantly disincentivize the supply side to forward shifting the extraction decision to the present period, over-signalling climate commitments may also favour this dynamic and increase cumulative resource extractions (*iii*); giving an international dimension to carbon regulation by involving multiple regions in a single partnership, and thus avoids carbon leakages. Without such a multi-scaled framework, polluting firms may move their production from a country with stringent policies, to a country with more lenient ones, or outsource the most carbon-intensive stages of the supply chains to other provisioners located outside of the zone regulated by the environmental agreement (*iv*).

All in all, it becomes obvious that the pattern of PCEU is affected by many other factors that should be taken into consideration by future studies (energy efficiency and intensity, sources of energy, environmental legislation). This constitutes the limit of current study which will be addressed in future research where alfa and beta-convergence might be also analysed for providing a better and holistic picture and generating valuable policy recommendations for the relevant stakeholders. Here, we underline the existence of some works [86–92] which employed Machine Learning (ML) methodologies derived from Artificial Intelligence (AI) and opened fruitful research directions on neighbouring topics. But there is left for future research. For instance, Magazzino et al.) combined the KOF Globalization Index, a GMM econometric methodology and a Long-Short-Term Memory (LSTM) algorithm as part of a three-stage procedure and showed that the latter approach may help reconciling dividing evidence reported by the formers [93].

## Abbreviations

AI: Artificial Intelligence
ANOVA: Variance Analysis Model
BNP: Bayesian Non-Parametric model
CIS: Commonwealth Independent States
CUSUM: Cumulative Sum
DDA: Distribution dynamics analysis
ICC: Intra Class Correlation coefficient
LSTM: Long-Short-Term Memory
MC: Monte-Carlo
ML: Machine Learning
N-L test: Non-Linearity test
NPA: Non-Parametric Approach
OLS: Ordinary Least Squares estimator
PA: Parametric Approach
PCEU: Per Capita Energy Use
PF-TYA: panel Fourier Toda-Yamamoto approach
PSCCM: Phillips-Sul club convergence method
PURT with SB: Panel Unit Root Test with Structural Break
QR: Quantile Regressions
RALS-LM: Lagrange Multiplier and Residual Augmented Least Squares-Lagrange Multiplier
SB: Stick Breaking
SCI: Subsampling Confidence intervals
SDGs: Sustainable Development Goals
S-GMM: System-Generalized Method of Moments
SPA: Semi-parametric Approach
SPSM: Sequential Panel Selection Method
UURT: Univariate Unit Root Test
